# mHealth Physical Activity and Patient-Reported Outcomes in Patients With Inflammatory Bowel Diseases: Cluster Analysis

**DOI:** 10.2196/48020

**Published:** 2024-09-24

**Authors:** Ashley C Griffin, Lucas Mentch, Feng-Chang Lin, Arlene E Chung

**Affiliations:** 1 VA Palo Alto Health Care System Palo Alto, CA United States; 2 Department of Medicine Stanford University School of Medicine Stanford, CA United States; 3 Department of Statistics University of Pittsburgh Pittsburgh, PA United States; 4 Department of Biostatistics University of North Carolina at Chapel Hill Chapel Hill, NC United States; 5 Department of Biostatistics and Bioinformatics Duke University School of Medicine Durham, NC United States

**Keywords:** inflammatory bowel diseases, patient-reported outcome measures, cluster analysis, wearable electronic devices, medical informatics, mHealth, mobile health, physical activity, bowel disease, psychosocial, smartphone, wearables, mobile phone

## Abstract

**Background:**

Regular physical activity is associated with improved quality of life in patients with inflammatory bowel diseases (IBDs), although much of the existing research is based on self-reported data. Wearable devices provide objective data on many rich physical activity dimensions including steps, duration, distance, and intensity. Little is known about how patients with IBDs engage in these varying dimensions of exercise and how it may influence their symptom and disease-specific patient-reported outcomes (PROs).

**Objective:**

This study aims to (1) cluster physical activity patterns from consumer-grade wearable devices and (2) assess the relationship between the clusters and PROs in patients with IBDs.

**Methods:**

We conducted a cross-sectional and longitudinal cohort study among adults with IBDs in the Crohn’s and Colitis Foundation IBD Partners cohort. Participants contribute physical activity data through smartphone apps or wearable devices in a bring-your-own-device model. Participants also complete biannual PRO questionnaires from the Patient-Reported Outcomes Measurement Information System short forms and IBD-specific questionnaires. K-means cluster analysis was used to generate physical activity clusters based on 3 key features: number of steps, duration of moderate to vigorous activity (minutes), and distance of activity (miles). Based on the clusters, we conducted a cross-sectional analysis to examine differences in mean questionnaire scores and participant characteristics using one-way ANOVA and chi-square tests. We also conducted a longitudinal analysis to examine individual cluster transitions among participants who completed multiple questionnaires, and mean differences in questionnaire scores were compared using 2-tailed paired sample *t* tests across 6-month periods.

**Results:**

Among 430 participants comprising 1255 six-week physical activity periods, we identified clusters of low (33.7%, n=423), moderate (46%, n=577), and high (20.3%, n=255) physical activity. Scores varied across clusters for depression (*P*=.004), pain interference (*P*<.001), fatigue *(P*<.001), sleep disturbance (*P*<.001), social satisfaction (*P*<.001), and short Crohn Disease Activity Index (*P*<.001), with those in the low activity cluster having the worst scores. Sociodemographic characteristics also differed, and those with low physical activity were older (*P*=.002), had higher BMIs (*P*<.001), and had longer disease durations (*P*=.02) compared to other clusters. Among 246 participants who completed at least 2 consecutive questionnaires consisting of 726 questionnaire periods, 67.8% (n=492) remained in the same cluster, and only 1.2% (n=9) moved to or from the furthest clusters of low and high activity across 6-month periods.

**Conclusions:**

For patients with IBDs, there were positive associations between physical activity and PROs related to disease activity and psychosocial domains. Physical activity patterns mostly did not fluctuate over time, suggesting little variation in exercise levels in the absence of an intervention. The use of real-world data to identify subgroups with similar lifestyle behaviors could be leveraged to develop targeted interventions that provide support for psychosocial symptoms and physical activity for personalized IBD care.

## Introduction

### Background

Inflammatory bowel diseases (IBDs), which include Crohn disease (CD) and ulcerative colitis (UC), are chronic intestinal disorders of the gastrointestinal tract. IBDs are characterized by cycles of active and dormant states of inflammatory immune response with symptoms such as abdominal pain, diarrhea, or fatigue [1]. Physical symptoms are frequently accompanied by stress, anxiety, depression, or diminished quality of life and may be exacerbated by common immunosuppressive therapies or corticosteroid treatments [2]. Supporting patients with IBDs to directly report their symptoms or functional status through patient-reported outcome (PRO) measures provides an important clinical end point to understanding the burden of the disease [3,4]. Physical activity may improve these physical and psychosocial symptoms, although there may be variations in inflammation related to the multidimensions of activity such as duration, frequency, or intensity [1,5]. For example, moderate physical activity interventions, including cardiovascular training, strength training, and yoga, have demonstrated generally positive effects on symptoms and inflammation [5-9]. However, high-intensity or extended durations of exercise can lead to inflammation and exacerbate gastrointestinal symptoms [6,10]. Examining these patterns of physical activity relative to PROs has not been well-studied but may be important to inform strategies for physically inactive subgroups of patients with IBDs.

Wearable devices provide objective mobile health data on physical activity, which include many rich dimensions of activity (ie, steps, distance, duration, frequency, intensity, and gait speed). Estimates indicate that up to 45% of the US population owns a wrist-worn wearable device [11,12]. Few studies have used data from wearables to assess physical activity in patients with IBDs, as much of this research is based on self-reported physical activity questionnaires [1]. Early findings using wearable devices suggest an association between physical activity and biomarkers for inflammation and disease activity [13,14]. Digital phenotyping, defined by Torous et al [15], is the “moment-by-moment quantification of the individual-level human phenotype in situ using data from personal digital devices.” Within the context of physical activity, digital phenotyping has been valuable in distinguishing different activity types from high-volume, heterogeneous wearable device data [16]. Prior studies have used clustering methods, such as K-means, to derive physical activity phenotypes, including “weekend warriors,” “busy bees,” “drivers,” “insufficiently active,” “cardio active,” and “deconditioned” [16-20]. Quantifying these physical activity behaviors can reveal unique lifestyle characteristics and insights into the disease’s impact on health status and functioning. Given the complex pathophysiology and heterogeneity of IBDs [21], accounting for individual variability in lifestyle behaviors is critical to inform personalized IBD care.

Little is known about whether there are physical activity phenotypes for patients with IBDs, yet identifying groups with similar physical activity patterns could facilitate tailoring lifestyle recommendations that enhance support for IBD. The use of real-world wearable device data provides opportunities to objectively understand the relationship between physical activity and the burden of disease. The Precision Visualization and Statistical Approaches (VISSTA) for patient-generated health data to enable precision medicine study focuses on developing health recommendations for lifestyle behaviors to improve health outcomes (ie, symptoms and quality of life) [22]. In this substudy of Precision VISSTA, we sought to identify physical activity phenotypes from consumer-grade wearable devices and examine the relationship between these phenotypes and PROs to support precision IBD care.

### Objectives

The objectives of this study were to (1) identify clusters of physical activity patterns and (2) evaluate the associations between physical activity and PROs (disease activity and quality of life domains) in patients with IBDs.

## Methods

### Study Design and Setting

We conducted a cross-sectional and longitudinal cohort study among participants in the Crohn’s and Colitis Foundation (CCF) IBD Partners study, which is an internet-based cohort of adults (18 years and older) living with IBD [23]. Participants in the CCF IBD Partners study have access to a portal to sync physical activity tracking smartphone apps or wearables (eg, Fitbit and Garmin) through a bring-your-own-device model [23,24]. Biannual questionnaires are completed for the PROMIS (Patient-Reported Outcomes Measurement Information System) domains of depression, anxiety, pain interference, sleep disturbance, satisfaction with social roles and activities, and fatigue. Disease activity questionnaires specific to the type of IBD (short Crohn Disease Activity Index [SCDAI] and Simple Clinical Colitis Activity Index [SCCAI]) are also completed biannually. This study followed the STROBE (Strengthening the Reporting of Observational Studies in Epidemiology) to report observational studies (Multimedia Appendix 1) [25].

### Ethical Considerations

This study was reviewed and approved by the institutional review board of the University of North Carolina at Chapel Hill (#17-2148). All individuals provided informed consent to participate in the IBD Partners parent study, and the data used for this study were considered secondary data analysis as all data were deidentified to protect privacy and ensure confidentiality. Separate consent was not required for this current study. A description of the data management system and establishment of IBD Partners (previously CCFA Partners) were previously reported.

### Participants

Participants were included in this analysis if they were enrolled in the CCF IBD Partners study between 2011 and 2020, completed at least one PROMIS and disease activity questionnaire, and contributed at least 50% of nonconsecutive or consecutive wearable device data within the 6 weeks before questionnaire completion. Participants could be included in multiple time periods if they completed more than one questionnaire. Approximately 20% (n=113) of the 543 participants who completed a questionnaire did not have sufficient activity data and were excluded. Thus, a total of 430 participants were included in our final analytic sample.

### Measures

PROMIS short forms were completed for each of the 6 health-related quality of life domains above and scored using standardized T-scores with a population mean of 50 (SE 10) [26]. Higher T-scores represent more of the concept being measured (eg, more fatigue or more satisfaction with social roles and activities). The SCDAI assesses the severity of symptoms (ie, stool frequency, abdominal pain, and well-being) within the past 7 days [27]. SCDAI scores range from 0 to over 600 with scores <150 indicating clinical remission and ≥150 indicating active disease (mild activity: 150-219; moderate activity: 220-450; and severe activity: >450) [27]. UC or indeterminate colitis (IC) disease activity was measured using the SCCAI, which assesses the severity of symptoms (ie, frequency of bowel movement during the day and night, urgency of defecation, blood in stool, well-being, and extracolonic features) within the past 7 days [28]. SCCAI total scores range from 0 to 19. Scores <2.5 correlate with remission, whereas scores ≥2.5 correlate with active disease [29]. We also obtained sociodemographic and clinical characteristics of the cohort.

### Wearable Data Preprocessing

Physical activity features varied across device brands (eg, Fitbit, Garmin, and Under Armour), so matrices were created to illustrate the capabilities of each device. Features were standardized into minutes for duration and miles for distance. Tukey’s method was used to assess outliers, which were considered observations 1.5 times less or greater than the lower and upper quartile ranges, respectively [30]. Unrealistic values were removed (eg, activity duration of 24 hours). To determine the period of physical activity prior to completing a questionnaire to include in our analysis, Spearman’s correlation coefficients were calculated to measure the strength of the relationship between physical activity (steps) and disease activity (SCCAI and SCDAI). Coefficients were calculated at various weeks prior to completing a questionnaire (ie, weeks 1, 2, 4, 6, 8, 12, and 24) with at least 50% of days with data returned during the time period. The period with the highest correlation between steps and disease activity was selected, which was 6 weeks. During this 6-week period, the correlation coefficient for steps and SCDAI was –0.19 (*P*<.001) and –0.14 (*P*=.007) for SCCAI.

Features included in the analysis were daily averages for each day that activity was recorded for a participant: number of steps, duration of moderate to vigorous activity (minutes), distance of activity (miles), number of calories burned, number of days the device was used during weekdays, and number of days the device was used during weekends. Features were then averaged during the 6 weeks prior to each questionnaire timepoint per participant. We initially performed featuring scaling (*z* score standardization and min-max normalization), which had little effect on the quality of clustering. Thus, the raw preprocessed data were used in the clustering.

K-means algorithm or Lloyd’s method [31], which is a simple and widely used algorithm that organizes data into nonhierarchical groups, was iteratively run using combinations of these features. The quality of clusters was evaluated using silhouette coefficients, which measure the amount of cohesion and separation within and among clusters [32,33]. Ranging from –1 to 1, higher coefficients indicate better-defined clusters [32,33]. The combination of features that produced the highest average silhouette coefficient was selected. This included 3 features: number of steps, duration of moderate to vigorous activity (minutes), and distance of activity (miles). These features were moderate to highly correlated as demonstrated by Spearman correlation coefficients (ranging between 0.63 and 0.95).

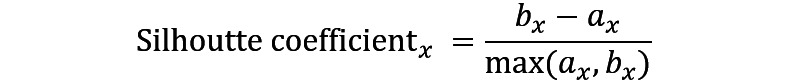



Here, *a_x_* is the average distance from an individual data point *x* to all data points within the same cluster and *b_x_* is the average distance from an individual data point *x* to all data points in the nearest cluster.

### Cluster Identification and Evaluation

K-means cluster analysis was then used to generate the physical activity clusters. K-means uses an iterative approach to assign each data point (*x*) to the closest centroid *c_i_* within each cluster *C_i_* by minimizing the average sum of squared Euclidean distance [32]. The equation below represents the K-means algorithm in which the sum of squared errors (SSE) or cluster scatter is minimized. K-means analyses were conducted using the Scikit-learn package in Python 3.7 [34]. The following parameters were used: number of clusters=3; initialization=k-means++; number of initializations=10; maximum iterations=300; tolerance=0.0001; random state=none. The algorithm was initialized 10 times with the centroids initially selected randomly (random state=none), and the initial centroid distances were optimized with k-means++. K-means++ places centroids to be generally distant from each other, which has been shown to improve the speed of convergence [35]. When the difference in the centroids across 2 consecutive iterations was lower than the tolerance level (0.0001), the algorithm converged and the iteration stopped.


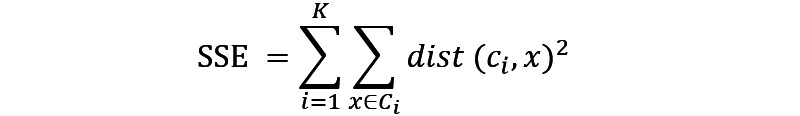


Here, *K* is the number of clusters, *x* is a data point that belongs to the cluster *C_i_*, *C_i_* is the *i*th cluster, *dist* is the standard Euclidean distance between two data points, and *c_i_* is the mean (centroid) of cluster *C_i_*.

**Figure 1:**
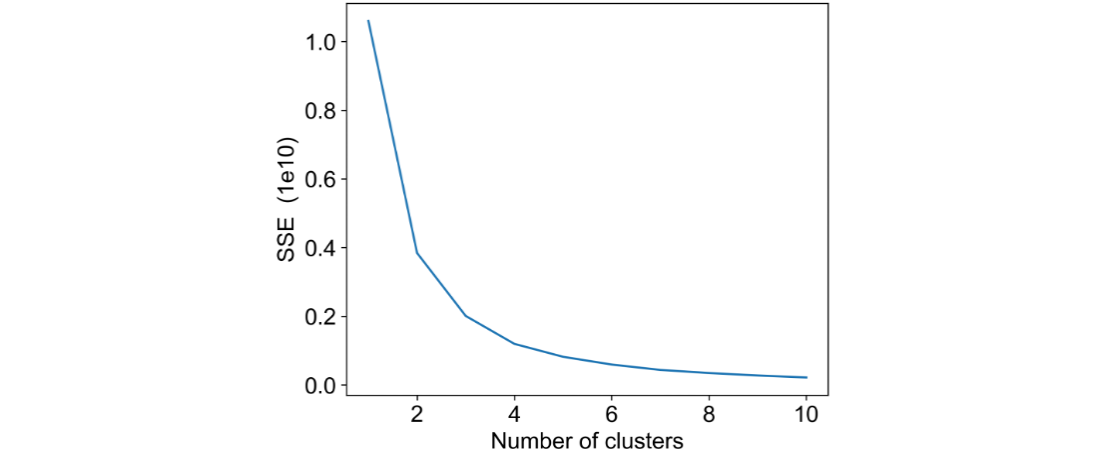
The SSE plot. SSE: sum of squared errors.

The number of clusters (K) was determined by calculating the SSE for different values of K. The value of K was selected where the change in SSE decreased and was becoming plateau, indicating additional clusters produced little value (“elbow point”). We iteratively evaluated the silhouette coefficients of 2 to 5 clusters and selected the model with the highest average silhouette coefficient, which was 3 clusters (Figure 1). Averages of silhouette coefficients were calculated for each cluster and the average of each of the 3 clusters. Once the clusters were identified, data within each cluster were assessed to determine a label that appropriately represented each cluster’s attributes [36].

### Association Between Physical Activity and PROs

Based on these clusters, we conducted two analyses: (1) a cross-sectional analysis (n=430) to assess differences in sociodemographics and PRO scores among the clusters, and (2) a longitudinal analysis (n=246) to examine mean changes in disease activity scores as participants moved to or from clusters among a subset of those who completed at least 2 consecutive biannual questionnaires. In the cross-sectional analysis, differences in sociodemographics and questionnaire scores among the clusters were assessed using one-way ANOVA tests and chi-square tests. Participants were included at multiple time periods if they had more than one 6-week physical activity period and completed the questionnaire. Including multiple observations per participant allowed us to examine the change in consecutive questionnaire scores as participants moved to or from clusters in our second analysis. In the longitudinal analysis, participants were grouped into categories related to staying in the same activity cluster or moving into another cluster across consecutive questionnaire time periods. For each move between clusters, mean differences in disease activity scores were compared using 2-tailed paired sample *t* tests. For all analyses, a *P* value <.05 was considered statistically significant.

## Results

### Participant Characteristics

The final analytic sample contained a total of 430 participants, of which 285 (66.3%) participants had CD and 145 (33.7%) participants had ulcerative or indeterminate colitis (Table 1). Participants were primarily women, White, and attained at least a college degree. On average, age was 42.1 (SD 13.6) years, BMI was 25.8 (SD 4.8) kg/m^2^, and duration of disease was 15.1 (SD 11.8) years. Nearly all had used corticosteroid medications (93.5%, n=400) or 5-aminosalicylic acid medications (86.3%, n=371) to treat their IBD. Participants in the sample completed an average of 3 (SD 2) questionnaires from 2015 to 2020. During the 6-week period prior to completing questionnaires, participants used their wearable device for an average of 37.3 (SD 6.2) days (88.9% of the 6-week period). This included approximately 89.7% (26.9 of 30) of weekdays and 86.7% (10.4 of 12) of weekends. The majority used a Fitbit device (86.3%, n=371). On average, participants took 7892.9 (SD 2752.7) daily steps, performed moderate to vigorous activity for 40.7 (SD 38.3) minutes, traveled 3.5 (SD 1.3) miles, and burned 520.7 (SD 170) calories during exercise.

**Table 1:** Sample characteristics^a^.

Characteristics		Value (N=430)
Age (years), mean (SD)		42.1 (13.6)
**Gender, n (%)**		
	Woman	318 (74)
	Man	112 (26)
**Race^b^, n (%)**		
	Asian	5 (1.2)
	Black	7 (1.7)
	White	394 (95.2)
	Other	8 (1.9)
**Ethnicity^b^, n (%)**		
	Not Hispanic or Latino	405 (97.1)
	Hispanic or Latino	13 (2.9)
**Education^b^, n (%)**		
	High school or less	21 (5)
	Some college	72 (17.1)
	College degree or more	327 (77.9)
BMI (kg/m^2^), mean (SD)^b^		25.8 (4.8)
**Smoking status, n (%)**		
	Ever	121 (28.1)
	Never	309 (71.9)
**Type of IBD^c^, n (%)**		
	Crohn disease	285 (66.3)
	Ulcerative or indeterminate colitis	145 (33.7)
Duration of disease (years), mean (SD)		15.1 (11.8)
**History of IBD treatments (ever used)^b^, n (%)**		
	5-Aminosalicylic acid medications	371 (86.3)
	Biologic therapies	301 (70.3)
	Corticosteroid medications	400 (93.5)
	Immunomodulators	282 (65.9)
**Device brand, n (%)**		
	Fitbit	371 (86.3)
	Garmin	43 (10)
	Jawbone	12 (2.8)
	Under Armour	4 (0.9)
Questionnaires completed, mean (SD)		3.0 (2.0)
Days device used (maximum of 42 days), mean (SD)		37.3 (6.2)
Days device used on weekdays (maximum of ~30 days), mean (SD)		26.9 (4.4)
Days device used on weekends (maximum of ~12 days), mean (SD)		10.4 (2.1)
Daily steps, mean (SD)		7892.9 (2752.7)
Daily moderate to vigorous duration in minutes, mean (SD)		40.7 (38.3)
Daily distance in miles, mean (SD)		3.5 (1.3)
Daily activity-related calories, mean (SD)		520.7 (170)

^a^Demographic and clinical characteristics are from the baseline questionnaire.

^b^Missing data: race=16; ethnicity=12; education=10; BMI=14; history of treatments for inflammatory bowel diseases=2 participants.

^c^IBD: inflammatory bowel disease.

### Clusters of Physical Activity Patterns

**Figure 2:**
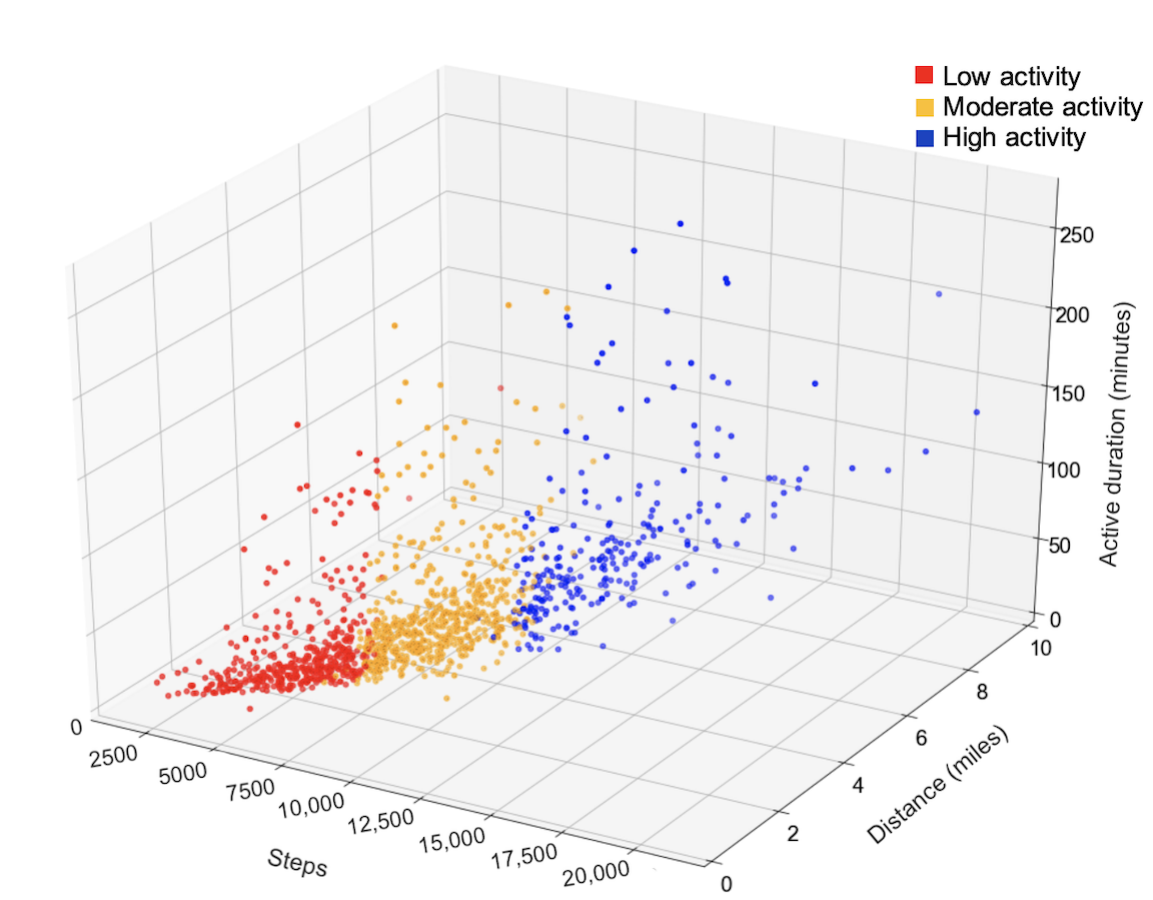
Physical activity clusters.

After examining data within each of the 3 clusters, we labeled the clusters in a way that was interpretable: low physical activity, moderate physical activity, and high physical activity (Figure 2). For the 430 participants, there were 1255 total 6-week time periods of which 423 (33.7%) time periods were classified as low activity, 577 (46%) time periods as moderate activity, and 255 (20.3%) time periods as high activity. A total of 146 of 430 participants were in multiple clusters where they had completed more than 1 questionnaire. Overall, clusters were moderately defined with an average silhouette coefficient=0.54 (Table 2). The quality of the clustering was highest in the low activity cluster (silhouette coefficient=0.60), suggesting that participants had the most similarities for steps, distance, and moderate to vigorous duration within this cluster. Those in the high activity cluster had the most variance in their levels of exercise, as indicated by the lowest silhouette coefficient (0.48) and highest standard deviations for steps, distance, and minutes of moderate to vigorous duration compared to the other clusters. Sociodemographic characteristics varied across clusters, and those in the low activity cluster were older (*P*=.002), had higher BMIs (*P*<.001), and had longer disease duration (*P*=.02) when compared to the other clusters. Participants in the low activity cluster also had higher proportions of those taking corticosteroids (*P*<.001) but lower proportions of those taking immunomodulators (*P*=.04).

**Table 2:** Physical activity cluster profiles.

Characteristics			Low activity (n=423)		Moderate activity (n=577)		High activity (n=255)		*P* value
**Evaluation**									
	Silhouette coefficient	0.60		0.52		0.48		—^a^	
**Features, mean (SD)**									
	Steps	5000.3 (1062.4)		8229.5 (1033.0)		12,319.4 (1899.6)		<.001	
	Distance (miles)	2.2 (0.6)		3.7 (0.7)		5.5 (1.1)		<.001	
	Moderate to vigorous duration (minutes)	21.3 (27.0)		40.6 (34.6)		73.0 (46.4)		<.001	
**Sociodemographics and clinical characteristics, mean (SD)**									
	Age (years)	45.8 (13.8)		43.5 (13.7)		42.1 (12.6)		.002	
	BMI (kg/m^2^)	27.8 (3.5)		25.6 (4.5)		23.6 (3.5)		<.001	
Duration of disease (years), mean (SD)			17.4 (12.3)		16.9 (12.0)		14.8 (10.0)		.02
**Current IBD^b^ treatment, n (%)**									
	5-Aminosalicylic acid medications	160 (37.8)		182 (31.5)		77 (30.2)		.06	
	Biologic therapies	252 (59.6)		328 (56.8)		141 (55.3)		.51	
	Corticosteroid medications	48 (11.3)		53 (9.2)		6 (2.4)		<.001	
	Immunomodulators	103 (24.3)		183 (31.7)		74 (29.0)		.04	

^a^Not applicable.

^b^IBD: inflammatory bowel disease.

### Association Between Physical Activity Clusters and PROs

#### Cross-Sectional Analysis

Across all PROs, patients in the low activity cluster had the worst scores, and those in the high activity cluster had the best scores (Table 3). Scores varied significantly across clusters on the level of depression (*P*=.004), pain interference (*P*<.001), fatigue *(P*<.001), sleep disturbance (*P*<.001), social satisfaction (*P*<.001), and SCDAI (*P*<.001). The largest mean differences between the low and high activity clusters for the PROMIS domains were scores for social satisfaction (5.0), fatigue (4.2), and pain interference (4.0). Mean SCCAI scores did not reach significance for patients with UC or IC, and those in the low and moderate activity clusters likely had some active disease activity during the 6-week period as scores ≥2.5 have previously demonstrated correlations with active disease [29]. Mean SCDAI scores were in remission (<150) in all clusters [27], and scores were highest in the low activity cluster.

**Table 3:** Patient-reported outcome scores across physical activity clusters.

	Low activity (n=423), mean (SD)	Moderate activity (n=577), mean (SD)	High activity (n=255), mean (SD)	*P* value
SCDAI^a,b^	133.3 (80.3)	117.0 (71.9)	102.2 (59.7)	<.001
SCCAI^a,c^	2.9 (2.1)	2.6 (1.8)	2.3 (2.5)	.20
PROMIS^d^ anxiety	50.4 (9.2)	49.3 (8.9)	49.2 (8.9)	.16
PROMIS depression	48.6 (8.3)	47.1 (7.8)	46.9 (7.3)	.004
PROMIS pain interference	50.7 (9.5)	48.5 (8.4)	46.7 (7.6)	<.001
PROMIS fatigue	54.9 (10.7)	51.5 (10.9)	50.7 (9.6)	<.001
PROMIS sleep disturbance	51.2 (7.5)	49.4 (7.7)	49.2 (7.8)	<.001
PROMIS satisfaction with social roles and activities	50.3 (9.7)	54.0 (9.1)	55.3 (9.1)	<.001

^a^Missing data: SCDAI (short Crohn Disease Activity Index)=44; SCCAI (Simple Clinical Colitis Activity Index)=78 questionnaires.

^b^SCDAI: short Crohn Disease Activity Index.

^c^SCCAI: Simple Clinical Colitis Activity Index.

^d^PROMIS: Patient-Reported Outcomes Measurement Information System.

#### Longitudinal Analysis

Among the sample of 430 participants who contributed wearable data and completed at least one questionnaire, 246 (57.2%) participants completed at least 2 consecutive disease activity questionnaires. In this subset of 246 participants (726 total questionnaires), 67.8% (n=492) did not change physical activity clusters during 6-month periods (Table 4). As expected, there were no significant changes in mean disease activity scores among those who did not move between clusters. For those who transitioned into another cluster, 15.8% (n=115) moved between (to and from) moderate and high activity, 15.2% (n=110) moved between low and moderate activity, and only 1.2% (n=9) moved between low and high activity clusters. Proportions of cluster movement were similar for patients with CD and UC or IC. There were significant associations between cluster movement and mean disease activity score for the 3 types of transitions. When patients with UC or IC transitioned from low to moderate activity clusters, disease scores decreased (*P*=.04). For CD, when patients moved from moderate to high activity or high to moderate activity clusters, disease scores decreased (*P*=.04) or increased (*P*=.02), respectively.

**Table 4:** Movement across clusters for consecutive inflammatory bowel disease activity scores.

Change in physical activity and cluster movement					n			Change in disease activity, mean (SD)			*P* value
**Ulcerative or indeterminate colitis (SCCAI^a^)**											
	**Improved**										
		Low → Moderate	21			–1.4 (2.4)			.04		
		Moderate → High	19			–0.4 (1.7)			.75		
		Low → High	0			N/A^b^			—^c^		
	**Reduced**										
		Moderate → Low	25			0.1 (1.2)			.74		
		High → Moderate	17			0.1 (2.1)			.91		
		High → Low	4			3.2 (3.8)			—^c^		
	**No change**										
		Low	51			0.1 (1.8)			.59		
		Moderate	76			–0.1 (1.3)			.55		
		High	38			–0.4 (1.8)			.18		
**Crohn disease (SCDAI^d^)**											
	**Improved**										
		Low → Moderate	26			–0.5 (82.8)			.34		
		Moderate → High	38			–25.3 (64.5)			.04		
		Low → High	1			0			—^c^		
	**Reduced**										
		Moderate → Low	38			7.6 (63.6)			.88		
		High → Moderate	41			20.6 (55.5)			.02		
		High → Low	4			78.8 (111.8)			—^c^		
	**No change**										
		Low	124			0.5 (67.9)			.94		
		Moderate	140			–6.3 (59.5)			.21		
		High	63			8.2 (69.8)			.35		

^a^SCCAI: Simple Clinical Colitis Activity Index.

^b^Not applicable.

^c^Paired sample *t* tests not calculated for n<5.

^d^SCDAI: Short Crohn Disease Activity Index.

## Discussion

### Principal Results

This study demonstrates how physical activity clusters can be generated from consumer-based wearable devices in patients with IBDs. Most participants (46%, n=577) were clustered in moderate activity, 33.7% (n=423) as low activity, and 20.3% (n=255) as high activity. Sociodemographic and clinical characteristics varied across clusters, and those with low activity were older, had higher BMIs, and longer disease durations. Our findings indicate positive associations between physical activity and health-related quality of life PROs in accordance with existing research [14,37]. Individuals in the low activity cluster had the worst scores across all PROs, and scores varied meaningfully on levels of depression, pain interference, fatigue, sleep disturbance, social satisfaction, and CD activity across clusters. UC activity indices did not vary significantly across clusters, which may be due to individuals in the low activity and moderate activity clusters who had some degree of active disease. These disease indices were aligned with individual’s IBD treatments, as there were higher proportions of those in the low activity cluster taking corticosteroid medications, which can be used as short-term treatment for IBD flares. Whereas, there were lower proportions of patients in the low activity cluster taking immunomodulators, which are usually used to maintain remission of IBD. We also found that those in the low physical activity cluster had the most homogeneity in exercise attributes, which suggests worse health status might hinder variations in exercise. These findings should be validated with other IBD cohorts to assess their reproducibility.

When we longitudinally assessed changes in physical activity and disease activity scores across 6-month periods, exercise patterns mostly did not fluctuate. Approximately 68% (n=492) of patients remained in their original cluster, and only 1% (n=9) of patients transitioned to or from the furthest clusters of low and high activity. This indicates that exercise levels may not vary to extremes over time. As expected, mean disease activity scores among patients who remained in the same cluster did not change over time. Despite prior literature suggesting the benefits of physical activity [1], the long 6-month longitudinal survey timepoints preclude any causal inferences. It is not possible to know, for example, whether increased physical activity levels reduced disease outcomes, or whether patients experiencing reduced symptoms were better able to exercise. Future studies that solicit more time points for symptom data from patients are needed to support this type of investigation.

### Implications for Health Care and Research

This research has several implications for the use of wearable devices and PROs for patients with IBDs. The use of real-world data to identify phenotypes with similar activity attributes could be leveraged to develop interventions that promote self-management and coping abilities, as these are important components in managing IBDs [38,39]. Previous studies have used wearables to personalize behavioral coaching strategies, which resulted in improvements in physical activity and clinical biomarkers (eg, lipids, hemoglobin A_1c_ levels) [40,41]. In this study, the low activity group was characterized by short durations of moderate to vigorous activity, steps, and distance. Interventions effective in improving sedentary behavior often use established behavior change techniques, including goal setting, self-monitoring, or social support [42]. We found the greatest difference among PROMIS domains between the low and high activity groups for social satisfaction, and evidence suggests social support may improve psychological symptoms and self-management behaviors in patients with IBDs [38,43]. New models of patient-centered care have been proposed, such as the IBD specialty medical home, which involve multifaceted approaches focusing on social support, behavioral skills, and stress management techniques [44]. Providing outreach to patients with changes in health status or physical activity, which could be indicated by cluster transitions, could be an important aspect of personalized IBD care. Moreover, as new therapies are being developed, the ability to observe distinguishable physical activity phenotypes could reveal insights related to the impact of pharmacotherapies within clinical trials or at the point of care. However, if real-time data are used within clinical care or for just-in-time interventions, necessary protocols and validation strategies are needed to ensure accurate data are presented to patients and care teams in a meaningful and easily interpretable way [45]. Visual analytics and additional machine learning approaches are being developed and evaluated as part of the Precision VISSTA study.

Patient-generated health data also have promising potential to facilitate the detection of inflammation. Evidence suggests certain inflammatory responses may be able to be detected through physiological measurements or lifestyle characteristics from wearables, such as elevated heart rate, elevated skin temperature, or sleep deviations [46-48]. Very few studies have assessed the relationship between wearable physiological measurements and inflammatory responses in patients with IBDs [13,14]. Sossenheimer et al [13] found lower daily steps within the week prior to elevated inflammatory biomarkers (ie, C-reactive protein and fecal calprotectin), but did not find differences in resting heart rate. Wiestler et al [14] also found lower levels of physical activity in patients with active disease compared to those in remission, in addition to lower sleep efficiency. Given the vast amount of data from sensor devices, robust analytical pipelines are necessary to process, analyze, combine with other data streams, and derive actionable information from the data [49]. Unsupervised learning models, which do not require costly labeled data, are useful for partitioning large data sets into smaller groups of related information. These related subgroups have the potential to inform strategies for the detection or mitigation of inflammatory responses given the fluctuating symptoms and disease trajectories that vary by patient.

### Limitations

The sample was limited to 430 participants in the CCF IBD Partners internet-based cohort who synced their smartphone app or wearable device, which may not be representative of all patients with IBDs. Future work should include larger and more diverse samples to improve the generalizability of these findings. In addition, the sample was more physically active than the general population, with our sample taking approximately 7900 steps per day. It is estimated that the US population takes 4800 steps per day (~5000 steps worldwide) [50]. There are also several limitations to using consumer-grade wearable device data. Most brands do not make the details of their algorithms or firmware updates available, so there may be differences in hardware or sensors across brands and devices over time. Innate user differences may also exist, such as the location the device was worn (dominant vs nondominant hand), wear time, or accuracy of manually logged exercise, which cannot be verified in the existing data. In addition, our clustering approach was limited by the lack of gradient or differentiation between individuals once clusters were established, so it is possible that individuals with similar activity patterns could be in different clusters. Finally, the sparse nature of the 6-month longitudinal survey timepoints precludes causal relationships.

### Conclusions

Recognition of patterns and changes in lifestyle behaviors by leveraging real-world mobile health data supports opportunities to inform interventions. Unsupervised learning techniques facilitate the identification of these patterns from vast, multidimensional wearable data. Patients in the low physical activity cluster reported the worst health-related quality of life and disease activity (ie, depression, pain interference, fatigue, sleep disturbance, social satisfaction, and CD activity) compared to those in moderate and high activity clusters. Additional support for physical and psychosocial symptoms and exercise may be valuable for those low physical activity IBD subgroups. Future research should examine these findings among more diverse cohorts with more frequent PRO measurements and validate reproducibility.

## Appendix

Multimedia Appendix 1 STROBE (Strengthening the Reporting of Observational Studies in Epidemiology) checklist.

## Abbreviations

CCF: Crohn’s and Colitis Foundation
CD: Crohn disease
IBD: inflammatory bowel disease
IC: indeterminate colitis
Precision VISSTA: Precision Visualization and Statistical Approaches
PRO: patient-reported outcome
PROMIS: Patient-Reported Outcomes Measurement Information System
SCCAI: Simple Clinical Colitis Activity Index
SCDAI: Short Crohn Disease Activity Index
SSE: sum of squared errors
STROBE: Strengthening the Reporting of Observational Studies in Epidemiology
UC: ulcerative colitis

## Data Availability

The data sets analyzed during this study are not publicly available to protect the privacy of individuals who participated in the study. Data are available from the corresponding author upon reasonable request. IBD Partners supports research studies, and investigators interested in accessing the data and pursuing a study should register on their website [51].

## References

[1] Bilski J, Brzozowski B, Mazur-Bialy A, Sliwowski Z, Brzozowski T (2014). The role of physical exercise in inflammatory bowel disease. Biomed Res Int.

[2] Subramanian CR, Triadafilopoulos G (2016). Care of inflammatory bowel disease patients in remission. Gastroenterol Rep (Oxf).

[3] Kochar B, Martin C, Kappelman M, Spiegel BM, Chen W, Sandler RS, Long MD (2018). Evaluation of Gastrointestinal Patient Reported Outcomes Measurement Information System (GI-PROMIS) symptom scales in subjects with inflammatory bowel diseases. Am J Gastroenterol.

[4] Kappelman MD, Long MD, Martin C, DeWalt DA, Kinneer PM, Chen W, Lewis JD, Sandler RS (2014). Evaluation of the patient-reported outcomes measurement information system in a large cohort of patients with inflammatory bowel diseases. Clin Gastroenterol Hepatol.

[5] Eckert KG, Abbasi-Neureither I, Köppel M, Huber G (2019). Structured physical activity interventions as a complementary therapy for patients with inflammatory bowel disease—a scoping review and practical implications. BMC Gastroenterol.

[6] Bilski J, Mazur-Bialy A, Brzozowski B, Magierowski M, Zahradnik-Bilska J, Wójcik D, Magierowska K, Kwiecien S, Mach T, Brzozowski T (2016). Can exercise affect the course of inflammatory bowel disease? Experimental and clinical evidence. Pharmacol Rep.

[7] Ng V, Millard W, Lebrun C, Howard J (2007). Low-intensity exercise improves quality of life in patients with Crohn's disease. Clin J Sport Med.

[8] Klare P, Nigg J, Nold J, Haller B, Krug AB, Mair S, Thoeringer CK, Christle JW, Schmid RM, Halle M, Huber W (2015). The impact of a ten-week physical exercise program on health-related quality of life in patients with inflammatory bowel disease: a prospective randomized controlled trial. Digestion.

[9] Gatt K, Schembri J, Katsanos KH, Christodoulou D, Karmiris K, Kopylov U, Pontas C, Koutroubakis IE, Foteinogiannopoulou K, Fabian A, Molnar T, Zammit D, Fragaki M, Balomenos D, Zingboim N, Horin SB, Mantzaris GJ, Ellul P (2019). Inflammatory bowel disease [IBD] and physical activity: a study on the impact of diagnosis on the level of exercise amongst patients with IBD. J Crohns Colitis.

[10] Ho GWK (2009). Lower gastrointestinal distress in endurance athletes. Curr Sports Med Rep.

[11] (2018). The wearable life 2.0: connected living in a wearable world. PWC.

[12] About one-in-five Americans use a smart watch or fitness tracker. Pew Research Center.

[13] Sossenheimer PH, Yvellez OV, Andersen M, Pearl T, Jurdi E, Rubin DB, Mayampurath A, Rubin DT (2019). Wearable devices can predict disease activity in inflammatory bowel disease patients. Gastroenterology.

[14] Wiestler M, Kockelmann F, Kück M, Kerling A, Tegtbur U, Manns MP, Attaran-Bandarabadi M, Bachmann O (2019). Quality of life is associated with wearable-based physical activity in patients with inflammatory bowel disease: a prospective, observational study. Clin Transl Gastroenterol.

[15] Torous J, Kiang MV, Lorme J, Onnela J (2016). New tools for new research in psychiatry: a scalable and customizable platform to empower data driven smartphone research. JMIR Ment Health.

[16] Marschollek M (2013). A semi-quantitative method to denote generic physical activity phenotypes from long-term accelerometer data—the ATLAS index. PLoS One.

[17] Lee I, Sesso HD, Oguma Y, Paffenbarger RS (2004). The "weekend warrior" and risk of mortality. Am J Epidemiol.

[18] Metzger JS, Catellier DJ, Evenson KR, Treuth MS, Rosamond WD, Siega-Riz AM (2008). Patterns of objectively measured physical activity in the United States. Med Sci Sports Exerc.

[19] McConnell MV, Shcherbina A, Pavlovic A, Homburger JR, Goldfeder RL, Waggot D, Cho MK, Rosenberger ME, Haskell WL, Myers J, Champagne MA, Mignot E, Landray M, Tarassenko L, Harrington RA, Yeung AC, Ashley EA (2017). Feasibility of obtaining measures of lifestyle from a smartphone app: the myheart counts cardiovascular health study. JAMA Cardiol.

[20] Koffman LJ, Crainiceanu CM, Roemmich RT, French MA (2023). Identifying unique subgroups of individuals with stroke using heart rate and steps to characterize physical activity. J Am Heart Assoc.

[21] Verstockt B, Noor N, Marigorta U, Pavlidis P, Deepak P, Ungaro RC, Scientific Workshop Steering Committee (2021). Results of the seventh scientific workshop of ECCO: precision medicine in IBD-disease outcome and response to therapy. J Crohns Colitis.

[22] VISSTA: Visualization & statistical approaches for PGHD to enable precision medicine.

[23] Long MD, Kappelman MD, Martin CF, Lewis JD, Mayer L, Kinneer PM, Sandler RS (2012). Development of an internet-based cohort of patients with inflammatory bowel diseases (CCFA partners): methodology and initial results. Inflamm Bowel Dis.

[24] Chung AE, Sandler RS, Long MD, Ahrens S, Burris JL, Martin CF, Anton K, Robb A, Caruso TP, Jaeger EL, Chen W, Clark M, Myers K, Dobes A, Kappelman MD (2016). Harnessing person-generated health data to accelerate patient-centered outcomes research: the Crohn's and Colitis foundation of America PCORnet patient powered research network (CCFA partners). J Am Med Inform Assoc.

[25] von Elm E, Altman DG, Egger M, Pocock SJ, Gøtzsche PC, Vandenbroucke JP, STROBE Initiative (2008). The Strengthening the Reporting of Observational Studies in Epidemiology (STROBE) statement: guidelines for reporting observational studies. J Clin Epidemiol.

[26] Cella D, Riley W, Stone A, Rothrock N, Reeve B, Yount S, Amtmann D, Bode R, Buysse D, Choi S, Cook K, Devellis R, DeWalt D, Fries JF, Gershon R, Hahn EA, Lai J, Pilkonis P, Revicki D, Rose M, Weinfurt K, Hays R (2010). The Patient-Reported Outcomes Measurement Information System (PROMIS) developed and tested its first wave of adult self-reported health outcome item banks: 2005-2008. J Clin Epidemiol.

[27] Thia K, Faubion WA, Loftus EV, Persson T, Persson A, Sandborn WJ (2011). Short CDAI: development and validation of a shortened and simplified Crohn's disease activity index. Inflamm Bowel Dis.

[28] Jowett SL, Seal C J, Phillips E, Gregory W, Barton J R, Welfare M R (2003). Defining relapse of ulcerative colitis using a symptom-based activity index. Scand J Gastroenterol.

[29] Higgins PDR, Schwartz M, Mapili J, Krokos I, Leung J, Zimmermann E M (2005). Patient defined dichotomous end points for remission and clinical improvement in ulcerative colitis. Gut.

[30] Tukey JW (1977). Exploratory Data Analysis (Addison-Wesley Series in Behavioral Science). 1st Edition.

[31] Lloyd S (1982). Least squares quantization in PCM. IEEE Trans Inform Theory.

[32] Tan P, Steinbach M, Kumar V (2005). Introduction to Data Mining.

[33] Rousseeuw PJ (1987). Silhouettes: a graphical aid to the interpretation and validation of cluster analysis. J Comput Appl Math.

[34] Pedregosa F, Varoquaux G, Gramfort A, Michel V, Thirion B, Grisel O, Blondel M, Prettenhofer P, Weiss R, Dubourg V, Vanderplas J, Passos A, Cournapeau D, Brucher M, Perrot M, Duchesnay M (2011). Scikit-learn: machine learning in Python. J Mach Learn Res.

[35] Arthur D, Vassilvitskii S (2007). K-Means++: the advantages of careful seeding. http://ilpubs.stanford.edu:8090/778/1/2006-13.pdf.

[36] Reedy J, Wirfält E, Flood A, Mitrou PN, Krebs-Smith SM, Kipnis V, Midthune D, Leitzmann M, Hollenbeck A, Schatzkin A, Subar AF (2010). Comparing 3 dietary pattern methods—cluster analysis, factor analysis, and index analysis—with colorectal cancer risk: The NIH-AARP diet and health study. Am J Epidemiol.

[37] Jones PD, Kappelman MD, Martin CF, Chen W, Sandler RS, Long MD (2015). Exercise decreases risk of future active disease in patients with inflammatory bowel disease in remission. Inflamm Bowel Dis.

[38] Kamp KJ, West P, Holmstrom A, Luo Z, Wyatt G, Given B (2019). Systematic review of social support on psychological symptoms and self-management behaviors among adults with inflammatory bowel disease. J Nurs Scholarsh.

[39] Conley S, Redeker N (2016). A systematic review of self-management interventions for inflammatory bowel disease. J Nurs Scholarsh.

[40] Price ND, Magis AT, Earls JC, Glusman G, Levy R, Lausted C, McDonald DT, Kusebauch U, Moss CL, Zhou Y, Qin S, Moritz RL, Brogaard K, Omenn GS, Lovejoy JC, Hood L (2017). A wellness study of 108 individuals using personal, dense, dynamic data clouds. Nat Biotechnol.

[41] Van Hoye K, Boen F, Lefevre J (2015). The impact of different degrees of Feedback on physical activity levels: a 4-week intervention study. Int J Environ Res Public Health.

[42] Stephenson A, McDonough SM, Murphy MH, Nugent CD, Mair JL (2017). Using computer, mobile and wearable technology enhanced interventions to reduce sedentary behaviour: a systematic review and meta-analysis. Int J Behav Nutr Phys Act.

[43] Regueiro M, Greer JB, Szigethy E (2017). Etiology and treatment of pain and psychosocial issues in patients with inflammatory bowel diseases. Gastroenterology.

[44] Regueiro MD, McAnallen SE, Greer JB, Perkins SE, Ramalingam S, Szigethy E (2016). The inflammatory bowel disease specialty medical home: a new model of patient-centered care. Inflamm Bowel Dis.

[45] Chung AE, Basch EM (2015). Potential and challenges of patient-generated health data for high-quality cancer care. J Oncol Pract.

[46] Li X, Dunn J, Salins D, Zhou G, Zhou W, Schüssler-Fiorenza Rose SM, Perelman D, Colbert E, Runge R, Rego S, Sonecha R, Datta S, McLaughlin T, Snyder MP (2017). Digital health: tracking physiomes and activity using wearable biosensors reveals useful health-related information. PLoS Biol.

[47] Radin JM, Wineinger NE, Topol EJ, Steinhubl SR (2020). Harnessing wearable device data to improve state-level real-time surveillance of influenza-like illness in the USA: a population-based study. Lancet Digit Health.

[48] Gadaleta M, Radin JM, Baca-Motes K, Ramos E, Kheterpal V, Topol EJ, Steinhubl SR, Quer G (2021). Passive detection of COVID-19 with wearable sensors and explainable machine learning algorithms. NPJ Digit Med.

[49] Dunn J, Runge R, Snyder M (2018). Wearables and the medical revolution. Per Med.

[50] Althoff T, Sosič R, Hicks JL, King AC, Delp SL, Leskovec J (2017). Large-scale physical activity data reveal worldwide activity inequality. Nature.

[51] (2023). IBD Partners.

